# Epidemiological characteristics of pediatric respiratory pathogens and their association with climate in Wuxi, Chongqing, China

**DOI:** 10.1016/j.nmni.2025.101656

**Published:** 2025-10-28

**Authors:** Caili Luo, Xingyu Wang, Na Zhou

**Affiliations:** a Department of Pediatrics, Wuxi People's Hospital, Wuxi County, Chongqing, China; b Laboratory Department, Wuxi People's Hospital, Wuxi County, Chongqing, China; c Department of Pediatrics, Bishan Hospital of Chongqing Medical University, Bishan County, Chongqing, China

**Keywords:** Pediatric respiratory infections, Climate-pathogen interactions, Seasonal epidemiology

## Abstract

**Background:**

Acute respiratory infections impose a disproportionate burden on pediatric populations, yet climate-pathogen interactions remain poorly characterized in rural China.

**Methods:**

We conducted a retrospective analysis of 7627 nasopharyngeal samples from children (<18 years) presenting with ARI symptoms at Wuxi People's Hospital (April 2023–March 2025). Six major pathogens--respiratory syncytial virus (RSV), influenza A/B (IAV/IBV), human rhinovirus (HRV), adenovirus (HAdV), and Mycoplasma pneumoniae (MP)--were detected by quantitative PCR. The association between meteorological factors and pathogen prevalence was examined.

**Results:**

HRV (22.18 %), IAV (18.54 %), and RSV (16.56 %) were the most prevalent pathogens, with RSV and HAdV showing higher hospitalization rates. Male children were more susceptible to all pathogens, particularly RSV (p = 0.024). Age-specific distributions revealed RSV dominance in infants (28 days-1 year) and toddlers (1–3 years), HRV and HAdV in preschoolers (3–6 years), and IAV and MP in school-aged children (6–18 years). Seasonal trends included year-round HRV and HAdV circulation, winter peaks for IAV and RSV, and an unexpected summer MP surge (26.88 % in June 2024). RSV exhibited an initial epidemic in 2023 (peak positivity: 52.27 % in April) with resurgence in October 2024. Climate analysis revealed temperature-dependent transmission: IAV/IBV correlated with colder temperatures. Notably, MP displayed atypical summer predominance in 2024, peaking at 26.88 % in June (25.2 °C, 77.3 % humidity).

**Conclusion:**

This study characterizes the epidemiological and climatic drivers of pediatric ARIs in Wuxi, China, underscoring pathogen-specific age distributions, seasonal anomalies, and climate interactions. The findings emphasize the need for sustained surveillance to monitor emerging respiratory threats and inform targeted interventions.

## 1 Introduction

Acute respiratory infections (ARIs) are a leading global cause of pediatric morbidity and mortality, disproportionately affecting low- and middle-income countries [1]. In 2016, ARIs ranked as the fourth leading cause of death worldwide, responsible for approximately 3 million fatalities, with acute lower respiratory tract infection like pneumonia and bronchiolitis driving most hospitalizations and deaths in young children [2].

Clinical manifestations of pediatric ARIs (e.g., cough, fever, and sore throat) are often nonspecific and overlap across diverse etiologies, complicating diagnosis and management [3]. While ARIs can be caused by bacterial, viral, or atypical pathogens. Viral pathogens—including respiratory syncytial virus (RSV), influenza (IFV), rhinovirus (HRV), adenovirus (HAdV), parainfluenza virus (HPIV), metapneumovirus (HMPV), and coronaviruses (HCoV)—account for the majority of cases [4]. These viruses display distinct epidemiological patterns, marked by high transmissibility, short incubation periods, and pandemic potential [5]. Notably, *Mycoplasma pneumoniae* (MP), an atypical bacterium, causes up to 40 % of community-acquired pneumonia (CAP) cases, particularly in school-aged children [6].

Accurate pathogen detection is critical for guiding treatment, curbing antibiotic misuse, and reducing healthcare burdens. Pathogen prevalence varies regionally due to climate, seasonality, and socioeconomic factors. For example, studies in China reveal temperature-dependent heterogeneity in influenza transmission, highlighting the need for localized public health strategies [7]. Air pollution and extreme weather (e.g., cold spells) may further exacerbate influenza-like illness, underscoring the complex interplay between environment and disease dynamics [8,9]. Such findings emphasize the importance of continuous surveillance to monitor shifts in pathogen prevalence and inform targeted interventions.

Wuxi County, a mountainous rural region in Chongqing (elevation: 139-2797 m), faces unique challenges due to its humid subtropical monsoon climate and persistent fog. Given their high prevalence and clinical relevance in pediatric ARIs in China, this study investigates the epidemiology of six major respiratory pathogens (RSV, IAV, IBV, HRV, HAdV, and MP) in Wuxi's pediatric population. Using multiplex qPCR, we analyzed 7627 nasopharyngeal samples from children with ARI symptoms (April 2023–March 2025). Our results elucidate local pathogen distribution, inform clinical and public health strategies, and address gaps in understanding climate-pathogen interactions in rural settings--a critical step toward targeted disease control.

## 2 Method

### 2.1 Study design and participants

A retrospective analysis was conducted on pediatric patients (outpatients and inpatients) presenting with respiratory symptomsat Wuxi People's Hospital between April 2023 and March 2025. Nasopharyngeal swabs from the patients served as experimental samples, and they were collected by specialized personnel from the respective laboratory departments. Inclusion: all children with fever, cough, rhinorrhea, or sore throat. Exclusion: only samples of insufficient quality for PCR analysis were excluded. All eligible samples received multiplex PCR testing. The laboratory testing was performed at Wuxi People's Hospital. The patients' essential clinical information was recorded and extracted from the hospital's electronic medical record system and no personally identifiable information of the patients was exported as part of this study. The Ethics Committee of Wuxi People's Hospital (Approval No. [2024–02] Lunshen [02]) approved this retrospective study and waived informed consent, as the research utilized exclusively anonymized archival data obtained from routine care.

### 2.2 Specimen collection and test

Within 24 h of admission, trained healthcare staff collected nasopharyngeal swab samples from participants. Total nucleic acids were extracted using the Natch-96B automated system (Sansure Biotech, China) and analyzed with the Six Respiratory Pathogen Nucleic Acid Detection Kit (Sansure Biotech; NMPA Registration No. 20213400256) on an MA-6000 qPCR instrument (YaRui, China). The multiplex assay simultaneously detects RSV, IAV, IBV, HRV, HAdV, and MP via pathogen-specific primers and probes. Each run included negative and positive controls, and an internal control (GAPDH) was used to monitor reaction validity. Inter- and intra-assay coefficients of variation were below 5 %.

Positive rate = Number of positive cases/total number of tested cases.

### 2.3 Meteorological data

Mean monthly temperature (°C), rainfall (mm), and humidity (%) were obtained from the Wuxi Weather Bureau.

### 2.4 Statistical analysis

Data collection and biostatistical analysis were performed using SPSS software. GraphPad Prism 8.0 software were utilized for data visualization. Descriptive statistics were used to describe the characteristics of the study sample and continuous data. The differences between mono-infection and coinfection were explored using the chi-square test. A T-test was used to compare the mean between groups. The variables with statistical significance were included. A *P* value < 0.05 was considered statistically significant.

## 3 Result

### 3.1 Demographic characteristics and sample overview

Between April 2023 and March 2025, 7627 pediatric respiratory samples were analyzed from Wuxi People's Hospital, comprising 3941 outpatients (51.67 %) and 3686 inpatients (48.33 %). The cohort showed a male predominance (56.14 %), with highest representation from preschool-aged children (3–6 years, 33.53 %) and toddlers (1–3 years, 26.56 %). Seasonal distribution revealed winter months (December–February) accounted for 41.77 % of cases, followed by spring (26.45 %)(Table 1).

**Table 1:** Demographic characteristics.

**Total**	7627
outpatient	3941(51.67 %)
inpatient	3686(48.33 %)
**Gender**	
Female	3345(43.86 %)
Male	4282(56.14 %)
**Age categories**	
Newborn(0-28d)	40(0.52 %)
Infants (28d-1y)	1211(15.88 %)
Toddlers (1-3y)	2026(26.56 %)
preschool children (3-6y)	2557(33.53 %)
School-age adolescents (6-18y)	1792(23.50 %)
**Season**	
Spring(from March to May)	2017(26.45 %)
Summer(from June to August)	1198(15.71 %)
Autumn(from September to November)	1226(16.07 %)
Winter(from December to February)	3186(41.77 %)

### 3.2 Pathogen detection rates

Among patients infected with at least one pathogen, human rhinovirus emerged as the most prevalent pathogen (HRV, 22.18 %), followed by influenza A (IAV, 18.54 %) and respiratory syncytial virus (RSV, 16.56 %). *Mycoplasma pneumoniae* (MP, 9.53 %) and adenovirus (HAdV, 9.00 %) demonstrated moderate prevalence, while influenza B showed the lowest detection rate (IBV, 4.78 %).

**Fig. 1:**
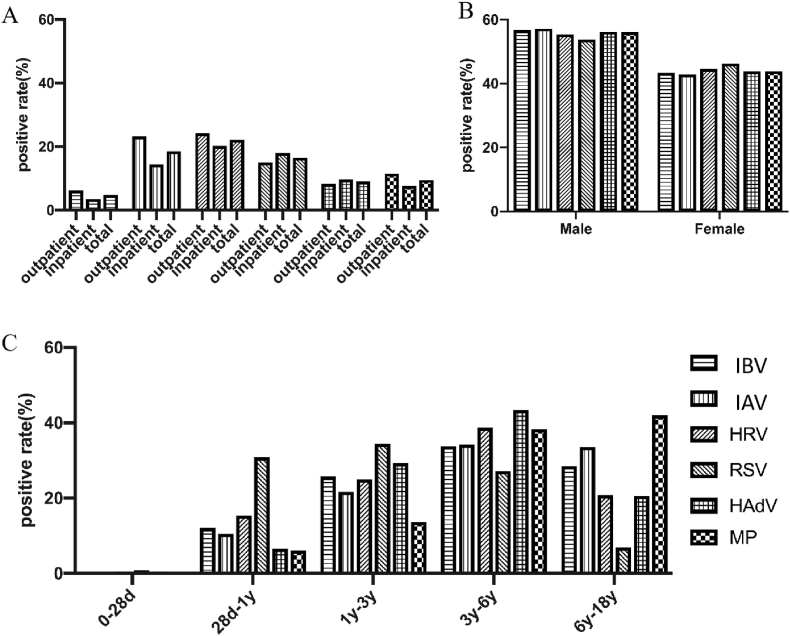
Distribution of single pathogen across different groups Distribution of a single pathogen in the outpatient/inpatient department. (B) Distribution of a Single Pathogen in different genders. (C) Distribution of a Single Pathogen at different age stages. Notes: influenza virus type B (IBV), influenza virus type A (IAV), rhinovirus (HRV), respiratory syncytial virus (RSV), adenovirus (HAdV), *Mycoplasma pneumoniae* (MP).

Further studied the different virus infection rates between out-patients and inpatients, we found that the incidence of viral infection in outpatients and inpatients were basically similar. Among outpatients, the pathogen with the highest positive rate was HRV, followed by IAV and RSV. Conversely, among inpatients, HRV had the highest positive rate, followed by RSV and IAV. Comparative analysis revealed similar pathogen hierarchies between outpatients and inpatients, though RSV and HAdV cases showed significantly higher hospitalization rates (p < 0.05) (Fig. 1A).

We compared male and female infections and found that boys were generally susceptible. The male prevalence was IBV at 56.66 %, IAV at 57.18 %, HRV at 55.44 %, RSV at 53.75 %, HAdV at 56.16 % and MP at 56.11 %. There was no significant difference in positivity rates between genders except RSV (*P* = 0.024) (Fig. 1B).

Age-stratified analysis identified distinct patterns: Neonates (0-28d) showed minimal infections, exclusively limited to RSV and HRV. Infants (28d-1y) and toddlers (1-3y) showed highest RSV susceptibility (peaking at 30.83 %), while preschool children (3-6y) exhibited predominance of HAdV (43.37 %) and HRV (38.73 %). School-age children (6-18y) demonstrated predominance of IAV (33.57 %) and MP (41.97 %). These age-related differences were statistically significant (p < 0.0001) (Fig. 1C).

### 3.3 Time distribution

**Fig. 2:**
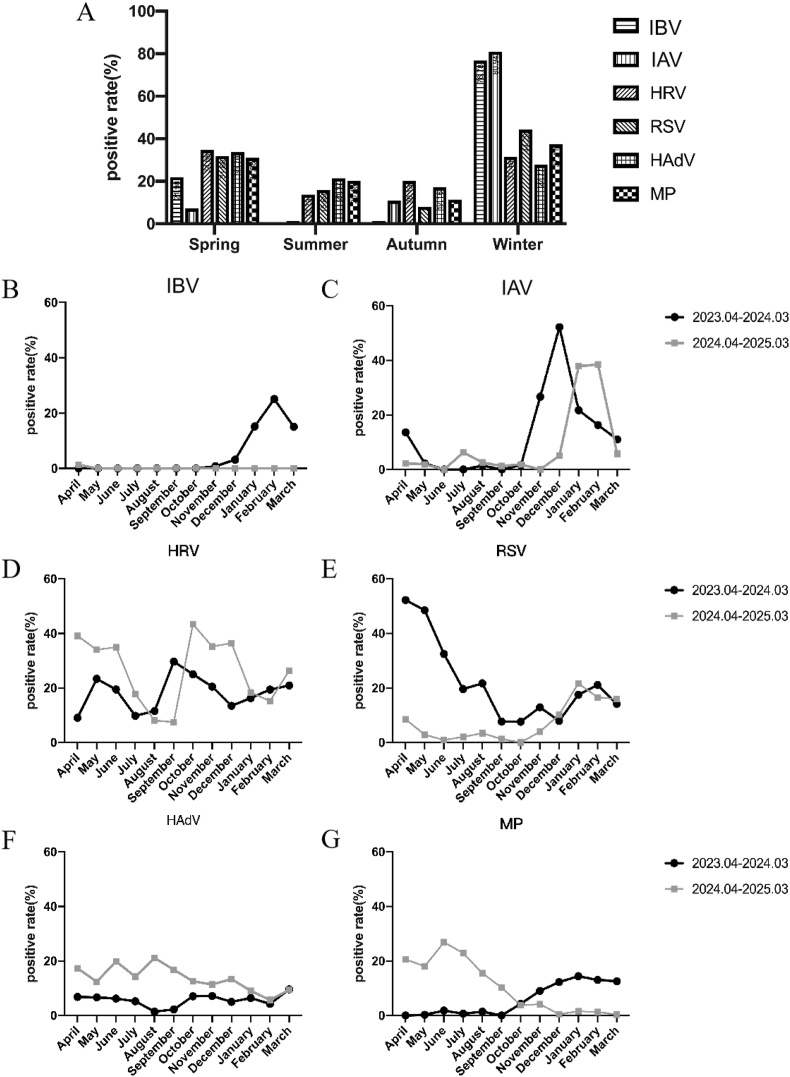
Seasonal and monthly distribution of pathogens detected positive from April 2023 to March 2025 Notes: influenza virus type B (IBV), influenza virus type A (IAV), rhinovirus (HRV), respiratory syncytial virus (RSV), adenovirus (HAdV), Mycoplasma pneumoniae (MP).

Seasonal trends revealed winter/spring predominance for most pathogens, especially IAV and IBV showed winter peaks (Fig. 2A).

Our longitudinal surveillance revealed distinct temporal patterns among respiratory pathogens. Influenza B (IBV) demonstrated a sharp but isolated outbreak, peaking at 25.09 % positivity in February 2024 before disappearing from subsequent surveillance (Fig. 2B). In contrast, influenza A (IAV) exhibited consistent winter seasonality, reaching 52.24 % prevalence in December 2023 and maintaining similar intensity (37.93 %) in January 2025, though its 2024 seasonal onset occurred approximately one month later than the previous year (Fig. 2C). HRV showed remarkable persistence throughout the study period, maintaining detection rates between 7.48 % and 43.40 % across all months without significant seasonal variation (Fig. 2D). RSV exhibited an epidemic pattern in 2023 (peak positivity 52.27 % in April) followed by resurgence in October 2024 (Fig. 2E). HAdV infections displayed intermittent activity, fluctuating between 1.45 % and 21.12 % prevalence after April 2024 without clear seasonal dominance (Fig. 2F). MP infections showed an unusual biphasic pattern: following stable baseline detection around 1 % before October 2023, rates rose significantly to 9.03–26.88 % during the October 2023–October 2024 period, culminating in an unexpected summer peak of 26.88 % in June 2024. MP exhibited a bimodal seasonal pattern, with peaks in the summer and winter of 2024 (Fig. 2G).

### 3.4 Viral coinfection patterns

**Fig. 3:**
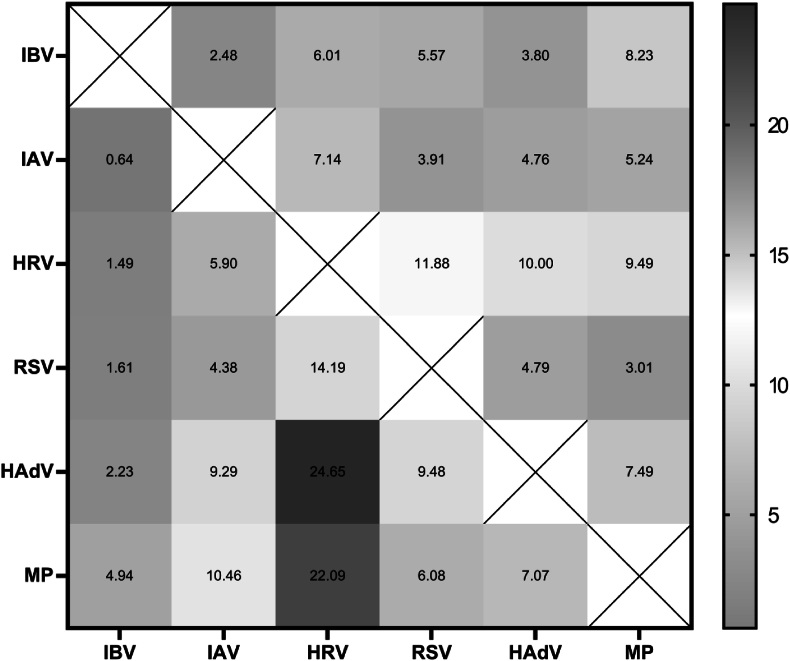
Coinfection pattern of respiratory pathogens in children with acute respiratory infections. The diagram illustrates the frequency of co-detection for pairs of pathogens among patients with acute respiratory infections. The analysis included all samples positive for at least one pathogen. The size and darkness of the circles are proportional to the relative frequency of each specific pathogen pair, calculated as the number of co-infected cases for that pair divided by the total number of co-infected cases identified in the study. Pathogen abbreviations: influenza B virus (IBV), influenza A virus (IAV), human rhinovirus (HRV), respiratory syncytial virus (RSV), adenovirus (HAdV), *Mycoplasma pneumoniae* (MP).

We analyzed coinfection patterns among the 7627 pediatric patients. Coinfection was defined as the simultaneous detection of two or more pathogens in a single sample. The relative frequency for each pathogen pair was calculated based on the total number of co-infection events. Our investigation revealed that human rhinovirus (HRV) emerging as the most frequent coinfection partner. While all studied pathogens demonstrated some capacity for concurrent infection, three specific combinations predominated: HAdV-HRV (24.65 %), MP-HRV (22.09 %) and RSV-HRV (14.19 %). These findings suggest that HRV's persistent year-round circulation may facilitate synergistic infections with seasonally variable pathogens (Fig. 3).

### 3.5 Climate correlations

**Fig. 4:**
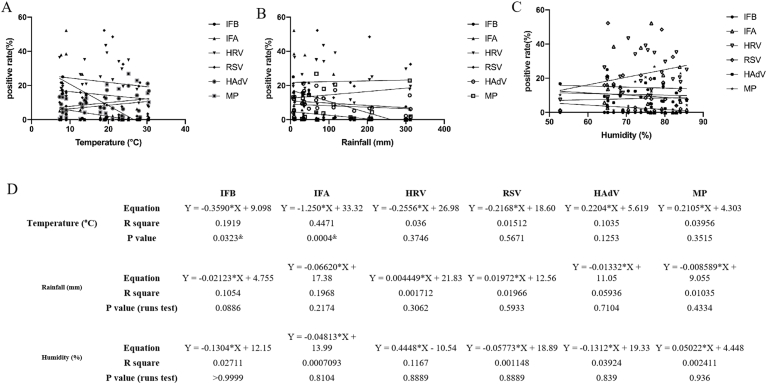
Analysis of the Correlation Between six major respiratory pathogens and Meteorology. Correlation with monthly average temperature. (B) Correlation with monthly rainfall. (C) Correlation with monthly average relative humidity. (D) Summary table of correlation coefficients and their statistical significance. Data are presented as correlation coefficients (r). A symbol (&) denotes a statistically significant correlation (p < 0.05). Pathogen abbreviations: influenza B virus (IBV), influenza A virus (IAV), human rhinovirus (HRV), respiratory syncytial virus (RSV), adenovirus (HAdV), *Mycoplasma pneumoniae* (MP).

IBV demonstrated complete temporal segregation, causing a discrete outbreak in February 2024 (25.09 % at 8.1 °C) while remaining undetectable during other winter seasons. IAV reached peak prevalence during the coldest months (December 2023: 52.24 % at 9 °C; February 2025: 38.55 % at 9 °C), showing strong negative correlation with temperature (r = −0.67, p = 0.0004). RSV presented the most dramatic epidemiological shift between study years. The expected spring 2023 epidemic (peak: 52.27 % in April at 18.9 °C) was followed by an atypical winter resurgence pattern in 2024–2025 (January peak: 21.72 % at 7.7 °C). This deviation from typical biennial RSV patterns may reflect post-pandemic ecological disturbances in viral transmission dynamics. HRV maintained consistent year-round circulation (7.48–43.40 %) but displayed amplified autumn activity, particularly in October 2024 (43.40 % at 19.3 °C). The pathogen's climate resilience contrasted sharply with other viruses, showing no significant correlation with temperature fluctuations (p = 0.18) or rainfall patterns. HAdV defied conventional seasonal expectations through its bimodal circulation pattern. Spring 2024 saw elevated activity (April–June: 17.33–19.89 % at 19.6–25.2 °C), followed by unexpected late summer peaks (August 2024: 21.12 % at 30.2 °C). Most remarkably, MP displayed unprecedented epidemiological behavior. While showing expected winter activity (January 2024: 14.40 % at 8 °C), the bacterium demonstrated extraordinary summer predominance in 2024, peaking at 26.88 % in June (25.2 °C, 77.3 %) (Fig. 4).

## 4 Discussion

From April 2023 to March 2025, we analyzed 7627 pediatric patients (<18 years) diagnosed with RTIs. HRV, IAV, RSV exhibited the highest detection rates, aligning with prior reports identifying these viruses as predominant contributors to pediatric RTIs [3]. Notably, HRV predominated in both outpatient and inpatient settings, followed by IAV and RSV, suggesting similar viral etiologies across healthcare tiers. The hospitalization rate of children with HAdV and RSV positive was higher. Similar to previous reports that RSV is prone to lower respiratory tract infections, which can lead to hospitalization [10]. The incidence of IFV in hospitalized patients is lower than that in outpatient patients. The reason may be that The IFV was shown to be less important in relating to pneumonia [11]. Unexpectedly, HRV was thought to cause only a “common cold”. Furthermore, these viruses in combination were responsible for greater annual morbidity than influenza viruses across all-age groups [3,12]. Our results underscore the need for preventive strategies against these prevalent viruses, including RSV prophylaxis and influenza vaccination in high-risk pediatric groups.

Notably, male children exhibited greater susceptibility to all pathogens studied, potentially due to immunological dimorphism and behavioral factors.

Respiratory virus susceptibility varies significantly across pediatric age groups, influenced by developmental, behavioral, and immunological factors. Our findings align with the established consensus that children under five are at the greatest risk for viral respiratory infections [13]. Consistent with global trends, RSV burden was highest in infants under one year of age prior to the COVID-19 pandemic, though a shift toward older children has been observed post-pandemic [7]. Our data confirm that RSV positivity peaks in infants (28 days–1 year) and toddlers (1–3 years), aligning with its known severity in young children [14,15]. Similar age shifts have been reported for other viruses, likely reflecting immunity gaps resulting from reduced exposure during the pandemic [16]. Furthermore, influenza virus (IAV) detection was most frequent in school-aged children, consistent with national and international surveillance data. These distinct age-specific patterns underscore the value of age-based profiling to guide clinical management and prevention strategies in settings with limited diagnostic capacity [17].

The year-round circulation with seasonal peaks for key pathogens in our study is consistent with established seasonal classifications of respiratory viruses from national surveillance data [18]. We observed a delayed onset of the influenza A (IAV) outbreak in 2024 compared to 2023, alongside a substantial reduction in influenza B (IBV) activity following its 2023 outbreak. The persistent global challenge of seasonal influenza, despite extensive interventions, underscores the role of viral evolution [19]. Genotyping confirmed IAV as the predominant subtype (58.3 %), consistent with prior reports [3]. Our study showed RSV exhibited an epidemic pattern in 2023 (peak positivity 52.27 % in April), this is not quite consistent with previous study [20]. But an unprecedented summer surge of RSV activity occurred in 2021 [7]. Longitudinal data (2017–2023) indicate significant disruptions to RSV seasonality due to the pandemic, including an absent winter peak in 2020–2021 and an atypical summer surge in 2021–2022, with a gradual return toward pre-pandemic patterns thereafter [21]. This aligns with global observations, such as a near-elimination of RSV detections in Western Australia and New Zealand following pandemic onset, highlighting the profound impact of public health interventions on viral circulation [22,23].

In our study, *Mycoplasma pneumoniae* (MP) displayed an unusual biphasic pattern, rising from a baseline of ∼1 % before October 2023 to a peak of 26.88 % by June 2024, resulting in a bimodal seasonal distribution with peaks in both summer and winter 2024. But the peak seasons of MP infection were fall and winter in the previous reports [24]. For example, the MP infection in Beijing peaked in the fall [25], and a survey of Xi'an showed that the peak infection was in the winter [25]. But there are also research reports in southern China, the outbreaks of MP infection tend to occur in summer or early fall [26] and the United States found that the rate of MP infection is the highest from August to November [27]. Our data from Wuxi, consistent with other southern reports, reveal earlier seasonal onsets compared to northern China, forming a clear latitudinal gradient in epidemic timing and underscoring the importance of region-specific surveillance.

Co-infections involving all viruses were detected at low frequencies, with HRV being the most common co-infecting agent alongside RSV, HAdV, and MP [3]. However, the clinical significance of PCR-based pathogen detection, such as distinguishing active HRV infection from carriage, remains challenging and necessitates integrating laboratory results with clinical presentation for accurate interpretation.

Notably, influenza A and B viruses exhibited distinct climatic sensitivities. While both showed negative associations with temperature and rainfall, their responses to humidity diverged markedly. Influenza A's positive correlation with relative humidity may reflect enhanced aerosol transmission in moist conditions, whereas influenza B's negative association could indicate greater dependence on contact transmission during dry periods. The extreme weather conditions of sustained low temperature and wet rain may have been important driving factors for the abnormal influenza A (H3N2) epidemic [28]. These differential climate preferences may contribute to the temporal segregation of influenza subtypes observed in our study. The climate associations of non-influenza pathogens revealed equally important patterns. RSV's negative correlations with both temperature and rainfall challenge conventional understandings of its epidemiology, potentially indicating strain-specific climate adaptations or changes in population immunity following the COVID-19 pandemic [29]. Similarly, MP's unexpected thrive in high-temperature conditions suggests possible evolutionary adaptations or climate-mediated changes in host susceptibility [29].

These findings highlight three key implications for public health: Firstly, the need for region-specific prevention strategies, such as tailoring influenza alerts to local climate. Secondly, the potential for pathogen-specific interventions, like using humidity to forecast influenza A/B dominance for vaccine planning. Finally, the necessity of adaptable surveillance systems to track pathogen ecological shifts under climate change.

Conclusions: This study delineates the epidemiological landscape of pediatric RTIs in Wuxi, China, highlighting climate-driven transmission patterns and diagnostic complexities. The emergence of MP as a summer pathogen and RSV's shifted seasonality sugguest ongoing ecological changes that demand close monitoring. The complex interplay between pathogens, host factors, and climate variables revealed by this study highlights the importance of maintaining robust surveillance systems to detect and respond to emerging respiratory threats in pediatric populations. Furture work should integrate multi-omics approaches to distinguish pathogenic from commensal viral loads and expand subtype-specific analyses.

Several limitations should be acknowledged. First, our study tested only six pathogens, potentially underestimating co-infection rates and overall disease burden from untested agents like pneumococcus, HCoV, PIV and HMPV. Second, our climate analysis did not incorporate air pollution data (PM2.5, NO_2_), known modulators of ARI risk. Third, the study period beginning in April 2023 limits our ability to assess pre-pandemic baseline patterns. Future studies should expand pathogen panels, integrate pollution monitoring, and extend surveillance periods to better characterize long-term trends.

## CRediT authorship contribution statement

**Caili Luo:** Writing – original draft, Investigation, Funding acquisition, Formal analysis. **Xingyu Wang:** Methodology, Formal analysis, Data curation. **Na Zhou:** Writing – review & editing, Writing – original draft, Project administration, Methodology, Investigation, Formal analysis, Data curation.

## Data availability statement

The original contributions presented in the study are included in the article; further inquiries can be directed to the corresponding authors.

## Consent for publication

Not applicable.

## Funding

Science and Technology Project of Wuxi County People's Hospital in 2024(Number:2024 keyan 05)

## Declaration of competing interest

The authors declare that they have no known competing financial interests or personal relationships that could have appeared to influence the work reported in this paper.
